# Dealing with Stigma: Experiences of Persons Affected by Disabilities and Leprosy

**DOI:** 10.1155/2015/261329

**Published:** 2015-04-15

**Authors:** Mimi Lusli, Marjolein B. M. Zweekhorst, Beatriz Miranda-Galarza, Ruth M. H. Peters, Sarah Cummings, Francisia S. S. E. Seda, Joske F. G. Bunders, Irwanto

**Affiliations:** ^1^Centre for Disability Studies, Selo Sumarjan Research Centre (SSRC), Faculty of Social and Political Sciences, Universitas Indonesia, Gedung H 6th Floor, Cubicle E, Kampus FISIP UI, Depok 16424, Indonesia; ^2^Athena Institute, Faculty of Earth and Life Sciences, VU University Amsterdam, De Boelelaan 1085, 1081 HV Amsterdam, Netherlands

## Abstract

Persons affected by leprosy or by disabilities face forms of stigma that have an impact on their lives. This study seeks to establish whether their experiences of stigma are similar, with a view to enabling the two groups of people to learn from each other. Accounts of experiences of the impact of stigma were obtained using in-depth interviews and focus group discussion with people affected by leprosy and by disabilities not related to leprosy. The analysis shows that there are a lot of similarities in impact of stigma in terms of emotions, thoughts, behaviour, and relationships between the two groups. The main difference is that those affected by leprosy tended to frame their situation in medical terms, while those living with disabilities described their situation from a more social perspective. In conclusion, the similarities offer opportunities for interventions and the positive attitudes and behaviours can be modelled in the sense that both groups can learn and benefit. Research that tackles different aspects of stigmatization faced by both groups could lead to inclusive initiatives that help individuals to come to terms with the stigma and to advocate against exclusion and discrimination.

## 1. Introduction

With an estimated total of 19,000 new leprosy cases in 2012, Indonesia has the third highest number of new leprosy cases after India and Brazil [1]. The provinces of East Java, West Java, Central Java, and South Sulawesi each report over a 1,000 new cases a year. Stigma has an important impact on the lives of people living with leprosy [2–5]. Studies have found that even people who are cured of leprosy can still remain trapped in the vicious circle of disease-impairments stigma and also face discrimination [6]. In 2008, the Transformasi Lepra Indonesia Foundation undertook research among people affected by leprosy who were living at home. Most of them had limited education, were unemployed, and lived in poverty. In addition, their families and communities had rejected them, as was apparent from their low participation in family and community events. Everyday discrimination was common; people affected by leprosy, for instance, were not allowed to use the same household utensils as other family members [7].

The meaning and connotation of the word stigma has varied considerably over the centuries. Today, the term refers to a personal attribute which marks a person as different from “normal people,” that is, “abnormal” with all its negative connotations, namely, exclusion from “normal” society [8]. Some studies have supported the idea that illnesses are stigmatized because of the limitations they entail and the negative social attitudes they generate [9, 10]. According to Fife and Wright (2000:51) “the specific nature of stigma associated with serious illnesses” depends on three elements: blaming the individual for the illness, the threat the illness represents to others, and the threat that it represents to individual competence. These elements are linked to a classical concept of stigma as an individual attribute, marking the difference between that person and those regarded as “normal” in a specific environment [8, 11]. Such a marker, or as Link and Phelan (2001) call it, a “label,” can affect the person who is stigmatized [12–15].

There has been considerable research on the effect of stigma on the lives of people with various diseases and disabilities. This has led to the development of a range of stigma-reduction interventions for various target groups worldwide [16–19]. Despite this research, few interventions have been developed for people affected by leprosy specifically. This raises the question whether the interventions developed for other target groups could also be effective in reducing the stigma faced by people who are affected by leprosy. In order to assess whether these interventions could have an effect, we first need to know whether the impact of stigma on the lives of people who are affected by leprosy and those who are affected by other diseases or disabilities not related to leprosy is similar or different. The research question addressed in this paper therefore is to determine the differences and similarities of the impact of stigma on the lives of people with leprosy and other diseases and disabilities living in a Cirebon, Indonesia.

In this paper we describe the first phase of the Stigma Assessment Reduction of Impact (SARI) Project in Cirebon District, Indonesia. The SARI project aims to assess the effectiveness of stigma reduction interventions in people affected by leprosy. The first phase of the project consisted of an exploratory study, in which we compared stigma experienced by people affected by leprosy with that of those who have visual and physical impairments not related to leprosy.

## 2. Theoretical Framework: Impact of Stigma

ILEP (2011) explored the impact of stigma on the lives of people who have health problems, such as leprosy The identified four domains in which stigma impacts on the lives of people: emotions, thoughts, behaviour and relationships. These domains are interconnected and manifest themselves in different degrees, at different moments, and in different contexts [20]. The first domain contains feelings such as “fear, grief, depression, shame, guilt, anxiety, low self-esteem, hopelessness and anger, or inability to express such feelings” (:7). The second domain describes the impact on thoughts in particular the “negative and pessimistic thoughts and beliefs about self, the world and the future” (:7). Emotions and thoughts influence the way people react and behave and can result in lack of confidence, avoidance, withdrawal from social life, and self-isolation. These elements are part of the third domain: behavior. Finally, the strength of the person's social support network and the attitudes of people in the network are important in the experience of stigma. The impact on the final domain, relationships, is described as “rejection, forced isolation and restricted social participation” (:7).

## 3. Method

The participants of this study were purposively selected. We tried to get a broad group in terms of sex, age, and marital status. Inclusion criteria were adults (between 20 and 65 years old) affected by leprosy and with disabilities (mental and intellectual disabilities were excluded). A qualitative approach was applied employing in-depth interviews (IDIs) and focus group discussions (FGDs). In-depth interviews were used to gain insights into the ways participants deal with stigma in their daily lives. Interviews lasted 45–60 minutes and took place in the homes of the participants. Fourteen participants (seven affected by leprosy and seven by disabilities) were interviewed on three occasions. First, the concept of stigma (or equivalent feelings and experiences) was discussed with the participants. Next, the four main domains of the theoretical framework (impact on emotions, thoughts, behaviour, and relationships) were used to explore the impact of stigma experienced by the participants.

The first focus group discussion was organized in the office of the SARI project and had 13 participants, seven persons affected by leprosy and six with disabilities. In this focus group we also focused on the four domains. From this focus group and the interviews we learnt that views on leprosy, disability, causes, and being cured strongly influenced the impact on thoughts. In addition, coping strategies were strongly impacting behaviour. Therefore, in the next two focus groups we elaborated on these concepts. The second focus group consisted of nine persons affected by leprosy and was held in SARIs office. The third focus group consisted of nine persons with disabilities and was held at the social office in subdistrict Lemah Abang.

The interviews and FGDs were recorded and transcribed. The data was analyzed by the first author of this paper, who is a person with a visual disability. Besides, for electronic data she used Braille to make notes and find themes, clusters, and patterns. The analyses focused on comparing the two groups: persons affected by leprosy and persons with disabilities in the impact of stigma faced in daily life on feelings, thoughts, behaviour, and relationships.

The participants gave their informed consent to be involved in the study and were also advised that the results would be published. They received no remuneration. Transport costs were covered when needed. The study was approved by the relevant offices, Ethics Committee of Atma Jaya University, Subdirectorate for Leprosy and Yaws, Ministry of Health, Public Health Office, West Java, and District Health Office, Cirebon District.

## 4. Results 

### 4.1. Stigma

Bahasa Indonesia does not have a precise equivalent of the word stigma. Participants used different, but largely common, terms to describe their feelings and experiences. Tables 1 and 2 present a list of different terms used by the study participants.

The participants discussed stigma, as understood in the terms given in Tables 1 and 2. An overview of the impact of stigma as discussed by the participants is presented in Table 3. The various elements are divided according to the four domains of the framework. In the sections below we elaborate on the differences and similarities between the two groups.

### 4.2. Impact on Emotions

Both groups expressed being identified as “different,” because of leprosy or their disabilities. They shared feelings of being shy, sad, confused, afraid, and powerless in the face of the stigma and discrimination they faced from the outside world. They also talked about feelings of guilt and about hiding from others, by staying at home for instance. Some voiced feelings of being a burden to their family. The participants explained that, since they believe what people say is true, they prefer to keep their feelings locked inside and not to share them with others:

*Why should I share my feelings with my family if I feel they do not care for my feelings? If I share them with people they will avoid (dijauhi) me even more. (Person affected by leprosy 1 IDI)*

The two groups were aware of the negative emotional impact that stigma plays in their everyday life. They expressed the belief that stigma is generated by their families and communities. A common argument was that they would like to overcome their feelings of self-stigma but found it impossible to do so because of strong social pressures:

*We are shy and have doubts about participating in daily life activities. We prefer to remain silent about our negative feelings, not because of us but because the community puts a label (cap) on us. (Person with disability 3 IDI)*

Although there are generally similarities between the perceptions and experiences of stigma of the two groups, the group affected by disabilities argued that they accepted themselves as having abnormal bodies but that they were not sick:

*I am ashamed of my hand. My hand is abnormal but I am not sick since I can still do my daily activities, manage a small business in front of my parent's house. But I am often shy and feel uncomfortable using my hand when I serve and interact with customers. (Person with disability 2 IDI)*

The group affected by leprosy see themselves as patients suffering from a disease: some continued seeing themselves this way even after the leprosy officer from the community health service had declared them cured. Apparently, the remaining deformities or pain were responsible for a persistent self-image as a “sick person.” As some participants explained,

*it cannot be that we are cured; we always feel pain in our muscles. We have skin patches and they never go away. (Person affected by leprosy 7 IDI)*

*See my left foot, it is crooked, it is abnormal, and it is sick. (Person affected by leprosy FGD1)*

*I always wonder when my left foot is going to be cured and become a normal foot again. (Person affected by leprosy 7 IDI)*

### 4.3. Impact on Thoughts

All participants were concerned about negative comments from others because of their different appearance: skin patches, physical deformity, and disabilities. They themselves, as well as their families and community members, regard their different appearance as something negative, labelling it, and marking/characterizing them by it. This may have a negative impact on their lives, limiting and restricting their social participation, making it difficult to assert their rights and satisfy their basic needs. For instance, both groups have faced challenges in finding a job. As one of them explained,

*when I attended an interview for recruitment the interviewer noticed the skin patch on my face. I told them I am cured from leprosy, but they did not trust me. I even showed them a formal letter from the community health services but they rejected me. (Person affected by leprosy IDI 1)*

Another participant added:

*I have to earn money for my children. Before I got leprosy, I worked as a cleaner, washing clothes, sweeping floors, and cooking. A few months ago, I was finally cured from leprosy, but nobody wants me to work in their house; people avoid me and ridicule (mengejik) me because of my crooked hand. (Persons affected by leprosy FGD 2)*

People with disabilities told similar stories. Indeed, both groups complained about experiencing rejection. One participant affected by disabilities expressed his frustration:

* Business communities have a prejudice regarding my impairment. When I applied for a job, they spontaneously label me saying I look as somebody who is looking for charity. (Persons affected by leprosy IDI 1) *

Another participant commented:

*When I showed my application, the employer did not look at it but he looked at my body and said “You are sick. We cannot accept employees like you. Our company does not have experience working with a person with impairment. You could be a burden for us.” (Person with disability FGD3)*

The participants reported dealing with their own negative thoughts provoked either by what people think and say about them or by what they think about themselves. The participants affected by leprosy explained that the lack of social relationships could be due to their status as sick people:

*We know we are sick because people have told us so. As sick persons, we have no friends, no hope, and no future. (Person affected by leprosy FGD1)*

*My disease is never going to be cured, so sick persons like me cannot work. (Person affected by leprosy IDI 4)*

One of the participants with disabilities said:

*We look different in our daily life, and people see our difference as something abnormal that might not be accepted wherever we are. (Person with disability FGD3) *

A participant with a visual impairment shared another experience:

*I am always avoided by people. When I try to participate in community activities, people say that I am an unfortunate person. (Person with disability FGD 3) *

Although all participants described feeling isolated, those affected by leprosy strongly see themselves in the role of a sick person. Persons with disabilities, on the other hand, seem to struggle more often with being seen as abnormal. The participants reported different understandings of leprosy, disability, causes, and being cured, as shown in Table 4.

### 4.4. Impact on Behaviour

All participants shared feelings of pessimism and a lack of motivation, as one of the participants affected by leprosy said:

*I do not know what I should do. I prefer to sit or walk around the house. If my family asks me for help, I help; if not, I usually just sit. (Person affected by leprosy FGD1)*

In particular, most of the participants affected by leprosy agreed: “We do better keeping our silence and staying away from people.” The participants affected by disabilities shared this attitude. As one person stated, “I do not want to do anything, I have given up. I prefer sitting or sleeping rather than starting any activity or doing something.” Stigma leads people to become passive and generates attitudinal barriers to undertaking action.

The group affected by leprosy said they prefer to keep silent and do nothing, as they feel that pursuing any activity will bring negative comments from the people around them:

*Keeping silent is better than doing something. Being labelled (cap) cannot be stopped and I cannot stop people labelling me. Meeting people, for me, means being insulted (dihina). (Person affected by leprosy IDI 6) *

Another participant added:

*When we [people affected by leprosy] are bored, we go to the kitchen and wash some dishes, but our family members shout at us and we get insulted (dihina). So we do not want to do it again, because doing activities is more isolating (dikucilkan). (Person affected by leprosy FGD 2)*

Some of the participants affected by leprosy justify their passiveness by their idea of being ill:

*We are ill. Going to the community health services, getting and taking our medicine regularly, meeting the health officers if we are in pain, and asking for medical treatment. That is enough. Just to do these actions is enough. (Person affected by leprosy FGD 2)*

People affected by disabilities face stigma, in which they feel it has to be accepted, although they also feel anger and sadness:

*I am sad about my abnormal body. People always tell me to see a doctor, to have medical treatment and stay at home. I am really angry but I cannot take any initiative. (Person with disability IDI 3) *

A participant with visual impairment expressed frustration:

*Self-isolation is the best choice. I cannot stand people staring (aneh) at me, feeling pity for my body. (Person with disability FGD 3) *

Both groups were aware that they will struggle with stigma throughout their lives and that they should adopt strategies to cope with it (see Table 5).

### 4.5. Impact on Relationships

The group affected by leprosy and the group affected by disabilities reported facing social exclusion due to stigma. They experience social barriers. Although the participants have said they consider themselves to be part of their families and society, they simultaneously feel rejected by them due to their appearance: skin patches, physical deformity, and other visible impairments.

The results show that social barriers were more evident when they were away from home: looking for a job, running a business, shopping, or just being outside their local neighbourhood. As shared by one of the participants affected by leprosy,

*once, I felt really disappointed when I met my neighbour. When I greeted him and offered him my hand, he rejected my hand without replying my greeting. Then he went inside his house and closed the door on me. He is a strange neighbour and what makes it even sadder is that he works in a community health service. (Person affected by leprosy IDI 2)*

Most of the participants affected by leprosy said they experience social barriers when they go outside the home and that they prefer to avoid any social contact when possible:

*If there are no urgent matters, we prefer staying at home. From our experience it is better to not make contact with people. People surrounding us expressly avoid us and they continually label us as patients with a contagious disease. (Person affected by leprosy FGD2)*

*For me, there is not any benefit in interacting with people. Many times I have tried to start social relations by smiling and saying “hi” to people, but they still look at me as an enemy. (Person affected by leprosy IDI 1)*

One of the participants with a visual impairment added:

*I am lonely although I live with my family. I feel like a member of my family but I am isolated by them. They discriminate me. They don't let me participate in preparing the food and cleaning the house. (Person with disability FGD 3)*

In their daily social life, both groups are aware of the stigma that is elicited when they try to establish social relations. People spontaneously reject them when they try to participate either in family life or in their respective communities.

For the group of people affected by leprosy, stigma may mean the end of all social encounters:

*It is impossible having social interaction with others. I am ill, I must be cured, get my hand back first, then I can go out. I believe people do not want to be friends with a sick person with a crawling hand like me. (Person affected by leprosy IDI 2)*

One person affected by disabilities argued that it is mainly the quality of the relationships that suffers. As one participant said,

*my right hand is amputated. When I want to shake the hand of another woman with my left hand she looks at me and takes a step back. Shaking hands with left hands is abnormal. I am disabled and this makes it difficult for me to build social relations with people who are normal and shake hands with their right hand. (Person with disability FGD 3) *

Furthermore, people affected by leprosy mentioned that their social relations are often restricted to the medical context, namely, with patients and health professionals:

*I am happy that the leprosy officer sometimes visits me at home, but I feel disappointed. He just comes to check whether I take the medicine regularly or not. I need people I can talk to. (Person affected by leprosy IDI 5)*

Predictably, persons affected by leprosy thought they would be better able to build social relationships once they were cured, while people affected by disabilities thought that expanding their social network depended on being “normal.”

### 4.6. A Perspective on Coping Strategies

Participants also shared some views showing that they can develop coping strategies to live with stigma. Some felt that the process of coming to terms with their condition made them more at ease with themselves:

*There is no choice but just to be patient. I try enjoying life. (Person with disability IDI 3)*

Sometimes participants have found that coming to terms with their condition is a good way to empower themselves. These participants show their desire to fulfil their needs such as getting food, medicines, or a job. Furthermore, persons affected by disabilities have struggled, trying to get access to work or to develop their own businesses. For instance, two participants with a physical impairment said:

*I am not sick and I run a business repairing electronic devices. I am abnormal, that is right, nevertheless I work as many other people do… Frequently, I challenge people by saying I am not sick, I am just abnormal. Even with this abnormal condition, I can still work and even earn money. (Person with disability FGD 3)*

*Basically I need to work. Having a job is my right. I must contact people for getting a job and earning money. I can ignore people who discriminate me. (Person with disability FGD 3)*

And a participant affected by leprosy noted:

*I have the rights to live so I need food and drink for my life. I need money to buy that, so I need to work. (Person affected by leprosy FGD 2)*

## 5. Discussion and Conclusions

This paper started with providing a detailed overview of how stigma is expressed in the language of Bahasa Indonesia, which might be a useful resource for other researchers and health professionals working with stigma in the Indonesian context. In this study, stigma experienced by people affected by leprosy and those with disabilities had negative impact on their emotions, thoughts, behaviours, and relationships, thus the four aspects of the framework [20]. Categorizing stigma experiences within these four domains was not always easy, as some experiences could fit in more than one domain.

Although the persons in this study differ in their type of illness and disability and the duration of impairment (from birth or attained at some point in their lives due to accident or illness), the experiences between the two groups demonstrate considerable similarity. Social exclusion and rejection come to the front in each domain and in both groups. Hence, these are likely two important concepts. In Indonesia, for disability similar experiences were described in the study of Kusumastuti et al. [21]. For leprosy, similar findings were provided in the study of Peters et al. [22] and Schuller et al. [23]. The study of Schuller et al., for instance, compared the experiences of women with disabilities due to leprosy and due to other reasons in Indonesia. They found that all women with disabilities experienced stigma, but those caused by leprosy were worse. It seems that stigma is most likely to be a generic phenomenon in this context. This is in line with van Brakel [24] who found that peoples' experiences with stigma and the consequences of stigma are remarkably similar among different health conditions and across cultures and countries.

Similarities in the experiences with stigma bring opportunities for interventions. People with different stigmatized conditions can perhaps benefit from the same interventions. As a group they can learn together about the challenges stigma brings. In addition, their thoughts on “difference” and behaviours as “social isolation” might improve by the interaction with a different group of people in similar circumstances. This was already happening, on a very small scale, with participants in our study. People affected by leprosy and with a disability met during the focus group discussion and due to the dialogues they seemed more able to respect differences and accept each other and one self.

The most notable difference in the experiences of stigma between the two groups is that people with leprosy were more likely to frame their condition as relating to “sickness,” even after they were cured, and those with disabilities were more likely to frame their condition as “abnormality.” The group affected by leprosy mostly understood leprosy as a contagious, chronic, and an incurable disease. Participants demonstrated negative attitudes that seem, in part, the result of false perceptions and limited knowledge about leprosy and disabilities, both in society as a whole and by the participants themselves. This was also described by others (e.g., Varkevisser et al. [25]). Such perceptions in people affected by leprosy constitute a form of self-stigmatisation. These findings illustrate the importance of interventions that can challenge deeply held beliefs and assumptions about, for instance, “difference,” “illness,” and “disability.” Counselling might be an example of such an intervention. Unfortunately, little is known about counselling in the field of leprosy. Floyd-Richard and Gurung conducted a pilot study with group counselling in Nepal that showed positive results [26].

One additional finding of this study is particularly interesting and relevant. While stigma has undoubtedly many harmful consequences, our study shows that some individuals have developed various coping strategies and are aware of their rights, found employment, and in this way contributed to the family income. From these people we can learn, in particular, when we develop interventions. Coping strategies have also been the focus in the study of Heijnders [27]. Heihnders specifically looked into health seeking and adherence to medication. Although their focus was different they in a similar fashion concluded that listening more carefully to those affected by leprosy is important if we want to improve leprosy services. The desire to contribute to the family income as expressed by participants in this study could be addressed by the development of socioeconomic interventions as, for instance, done by Ebenso et al. [28]. Skills people develop in such an intervention can help generate money and provide a living, which might make people around them view them more positively.

In conclusion, we argue that there are many similarities in the stigma experiences of persons affected by leprosy and persons with disabilities. The most important difference we found was that those affected by leprosy tended to frame their situation in medical terms, while those living with disabilities described their situation from a more social perspective. The similarities between the two groups and the coping strategies depicted by some persons offer opportunities for interventions. Research that tackles different aspects of stigmatization faced by people affected by leprosy and related impairments could lead to inclusive initiatives that help individuals to come to terms with the stigma and to advocate against exclusion and discrimination.

## Figures and Tables

**Table 1:** Terms related to the concept of stigma used by people affected by leprosy.

Indonesian	English equivalent
*Cap *	Label
*Tanda *	Mark
*Beda *	Difference
*Sakit *	Sick
*Menular *	Contagious
*Dikucilkan *	Isolated
*Dijauhi *	Avoided
*Sial *	Unfortunate
*Aneh *	Staring
*Dihina *	Insulted
*Mengejik *	Ridicule
*Diasingkan *	Excluded

**Table 2:** Terms related to stigma used by people affected by disabilities.

Indonesian	English
*Tidak normal *	Abnormal
*Beda *	Difference
*Rusak *	Broken
*Sakit *	Sick
*Merepotkan *	Burden
*Aneh *	Staring
*Menular *	Contagious
*Kasihan *	Pity
*Terbatas *	Limitation
*Kurang *	Incomplete
*Dipisahkan *	Separated
*Diasingkan *	Excluded

**Table 3:** Elements of impact by stigma divided by emotions, thoughts, behaviour, and relationships.

Emotions	Thoughts	Behaviour	Relationships
Afraid	Death	Moving away	Rejection
Worry	No hope	Passive	Separation
Mourning	No future	Self-isolation	Restriction in social participation
Shy	Isolated	Keep silent	Discrimination
Guilt	Feeling sick	Do not want to look for help	Stop relationships
Anxious	Not being accepted	Protected by family	Withdraw
Inferiority	Contagious	Stay at home	Reject invitation
Doubt	Avoided	Do not want to meet people	No contact
Angry	Burden	Hiding	No social role and responsibility
Sad	Limitation	Instability	No friends
Annoying	Belief in having been cured	Lying down	Difficult to get job
Confusion	Hope for change in the future	Be opened to listen to	Stand on their own
Hate	Get knowledge about the disease	Discipline	Keep trying
Patience		Accept people's help	Provoke encounters
Cheerful		Accept their condition and situation	Courage to meet doctors and leprosy officers

**Table 4:** Understandings of leprosy, disability, and cure.

People affected by leprosy		People affected by disabilities	
Indonesian	English	Indonesian	English
Leprosy		Disability	
*Rematik *	Rheumatic	*Bentuk tubuh berbeda, tidak normal *	Different form of body, abnormal
*Bercak *	Skin patch	*Rusak *	Broken
*Mati rasa *	Loss of sensitivity	*Tidak bisa digunakan *	Cannot be used
*Bengkok *	Crawling hands	*Cara beda, tidak normal *	Different way, abnormal
*Putus *	Falling apart fingers	*Palsu *	Prosthesis
*Luka *	Wound	*Katarak *	Cataract
*Pegallinu *	Pain	*Polio *	Polio
*Gatal *	Itchy	*Diperlakukan sebagai orang sakit *	Be treated as patients
*Kambuh *	Reaction	*Disuruh di rumah *	Asked to stay at home
*Ke puskesmas sebulan sekali ambil obat *	Once a month take medicine from the community health service	*Dilindungi karena sakit *	Protected due to illness
*Rutin minumobat *	Take medicine routinely	*Diabaikan karena tidak berguna *	Ignored due to uselessness
*Dinasehati terus *	Be continually advised	*Pakai alat (kruk, kursi roda, tongkat putih, etc.) *	Use device (wheelchair, crutch, white cane, etc.)

Causes			
*Kotor *	Dirty	*Virus *	Virus
*Guna-guna *	Magic	*Dari lahir *	From birth
*Kutukan *	Curse	*Keturunan *	Genetic
*Keturunan *	Genetic	*Kecelakaan *	Accident
*Alergy *	Allergy	*Tidak bisa sembuh dan sudah nasib *	Cannot cure, destiny
		*Dosa *	Sin

Cured			
*Sembuh berarti tidak ada bercak, pegal linu dll *	Cure means no skin patch, no pain, and so forth	*Tidak ada pengobatan *	No medical treatment
*Sembuh berarti stop minum obat *	Stop taking medicine	*Tidak perlu berobat, ke dokter, ke puskesmas *	No need to see doctor, no need to take medicine from community health service
*Diterima *	Be accepted	*Dipandang bila punya uang, kerja/usaha, status sosial, dekat dengan pemerintah dan tokoh masyarakat *	Be recognized if having money, job/business, social status, close with government and community leader
*Bisa beraktivitas (kerja, sekolah, ke pasar, ke sawah dll) *	Can perform activities (work, school, market, farm, etc.)		

**Table 5:** Strategies to cope with stigma.

People affected by leprosy		People affected by disabilities	
Indonesian	English	Indonesian	English
*Diam *	Silence	*Diam *	Silence
*Menjauhi *	Being far from people	*Cuwek *	Do not care/ignore
*Tidak berdekatan *	Not being close	*Di rumah *	Staying at home
*Menghindar *	Avoid	*Dengan teman sesame *	Being with peers
*Menolak ajakan *	Reject invitation	*Terlibat *	Participate in activities
*Berpindah *	Not moving	*Ke belajar dan berlatih *	Studying and training
*Pengobatan *	Medical treatment	*Menunjukkan kemampuan *	Showing capacities
*Minum obat *	Taking medicine	*Ambil bagian *	Taking part
*Dijelaskan *	Being advised	*Berjuang *	Fight
*Mengunjungi *	Visit people	*Punya hak *	Having rights
*Terlibat *	Participating in activities	*Bertahan *	Standing on their own
